# Research Progress and Application of Miniature CRISPR-Cas12 System in Gene Editing

**DOI:** 10.3390/ijms252312686

**Published:** 2024-11-26

**Authors:** Qiangbing Xuan, Junjie Wang, Yuanqing Nie, Chaowei Fang, Weihong Liang

**Affiliations:** College of Life Science, Henan Normal University, Xinxiang 453007, China; xuanqiangbing@163.com (Q.X.); nieyuanqing527@163.com (Y.N.); fangchaowei@htu.edu.cn (C.F.)

**Keywords:** miniature Cas12 protein, CRISPR-Cas, characteristic, gene editing, optimization strategy

## Abstract

CRISPR-Cas system, a natural acquired immune system in prokaryotes that defends against exogenous DNA invasion because of its simple structure and easy operation, has been widely used in many research fields such as synthetic biology, crop genetics and breeding, precision medicine, and so on. The miniature CRISPR-Cas12 system has been an emerging genome editing tool in recent years. Compared to the commonly used CRISPR-Cas9 and CRISPR-Cas12a, the miniature CRISPR-Cas12 system has unique advantages, such as rich PAM sites, higher specificity, smaller volume, and cytotoxicity. However, the application of miniature Cas12 proteins and the methods to improve its editing efficiency have not been systematically summarized. In this review, we introduce the classification of CRISPR-Cas system and summarize the structural characteristics of type V CRISPR-Cas system and the cleavage mechanism of five miniature Cas12 proteins. The application of a miniature CRISPR-Cas12 system in the gene editing of animals, plants, and microorganisms is summarized, and the strategies to improve the editing efficiency of the miniature CRISPR-Cas12 system are discussed, aiming to provide reference for further understanding the functional mechanism and engineering modification of the miniature CRISPR-Cas12 system.

## 1. Introduction

The CRISPR-Cas system is composed of Clustered Regularly Interspaced Short Palindromic Repeats (CRISPR) and CRISPR-associated sequence (Cas) that constitute a natural acquired immune system for prokaryotes to resist the invasion of exogenous DNA such as viruses, phages, etc. [1,2,3]. CRISPR-Cas can accurately induce a double-stranded break (DSB), and then, the double-stranded DNA (dsDNA) activate the endogenous DNA homology directed repair (HDR) and non-homologous end joining (NHEJ) repair mechanisms. Therefore, the CRISPR-Cas system can be used to knock out, insert, and modify genomes in animals, plants, and microorganisms, so as to achieve the purpose of gene editing [4,5,6,7].

CRISPR-Cas systems have a simple structure and are easy to operate, which can specifically recognize and cleave nucleic acid sequences at specific locations. Among them, CRISPR-SpyCas9 (*Streptococcus pyogenes*, 1368 aa) [8], CRISPR-LbCas12a (*Lachnospiraceae bacterium*, 1223 aa) [9], and CRISPR-AsCas12a (*Acidaminococcus* sp., 1307 aa) [10] are widely used as gene editing tools. However, some imperfections still exist, such as difficult delivery, single protospacer adjacent motif (PAM), and low editing efficiency of specific sites. Recently, several miniature Cas proteins with nucleic acid cleavage activity and a volume less than 800 aa have been continuously reported. These miniature Cas proteins belong to the Class 2 of type V CRISPR-Cas12 systems, including Cas12f (400–700 aa) [11], Cas12g (767 aa) [12], Cas12j (CasΦ, 700–800 aa) [13], Casλ (747 aa) [14], and Cas12n (400–700 aa) [15]. These natural miniature Cas proteins have low editing activity, which can be improved by guide RNA (gRNA), Cas protein optimization, and temperature treatment, and efficiently strategies have been successfully applied to genome editing of animals and plants.

Here, we systematically analyzed and compared the structural characteristics, cleavage mechanism, and application status of five miniature CRISPR-Cas12 proteins in microorganisms, animals, and plants. Furthermore, we discussed the methods to improve the editing efficiency of the miniature CRISPR-Cas12 protein, aiming for clearer understanding and better development and utilization of the CRISPR-Cas12 system.

## 2. Classification of the CRISPR-Cas System

**Figure 1:**
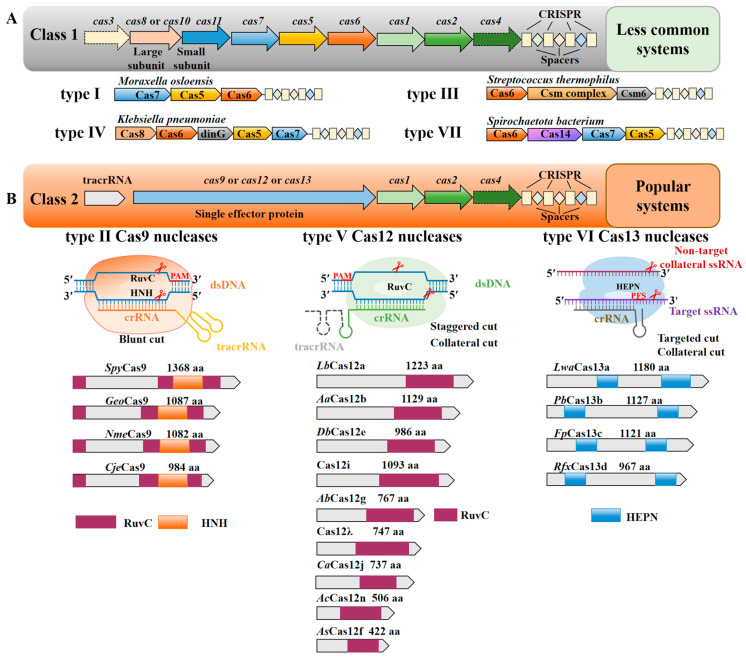
The classification and characteristics of the CRISPR-Cas system. (**A**) The general structures of the CRISPR-Cas loci in Class 1 and the typical protein structure diagram of each type. Genes are shown as arrows; the yellow rectangle and diamond represent CRISPR and spacers, respectively. (**B**) The general structures of the CRISPR-Cas loci in Class 2 and the typical protein structure diagram and cutting mechanism of each type. Genes are shown as arrows; the yellow rectangle and diamond represent CRISPR and spacers, respectively; Three irregular patterns represent three different nucleases; the deep red, orange, and blue rectangles represent the cleavage domain of the corresponding nuclease [4,26].

According to the different compositions of CRISPR sequence and Cas protein composition, the CRISPR-Cas system is divided into two classes and seven types. Among them, Class 1 consists of type I, type III, type IV, and type VII. The common feature is a multi-subunit effect complex composed of multiple Cas proteins, and only a small part has been developed as a gene editing tool [16,17,18,19,20] (Figure 1A). Class 2 includes type II, type V, and type VI, all of which are formed by a single large Cas protein and gRNA complex (Figure 1B). The protein encoded by type II is Cas9, an endonuclease guided by crRNA and tracrRNA, including two cleavage domains of Histidine-asparagine-histidine (HNH) and RNase H-like fold (RuvC) [21]. Among them, CRISPR-SpyCas9 is widely used in gene editing of animal and plant cells, opening up a new era of gene editing [22]. Unlike the CRISPR-Cas9 system, the type V CRISPR-Cas12 system requires only a single RuvC cleavage domain to effectively cleave dsDNA and single-stranded DNA (ssDNA), which greatly reduces the size of the CRISPR-Cas12 system [23]. The effector protein encoded by type VI is Cas13, which cleaves RNA and is widely used in RNA editing, imaging, and detection, as well as RNA-based gene therapy [24,25].

## 3. Characteristics of the Type V CRISPR-Cas System

**Figure 2:**
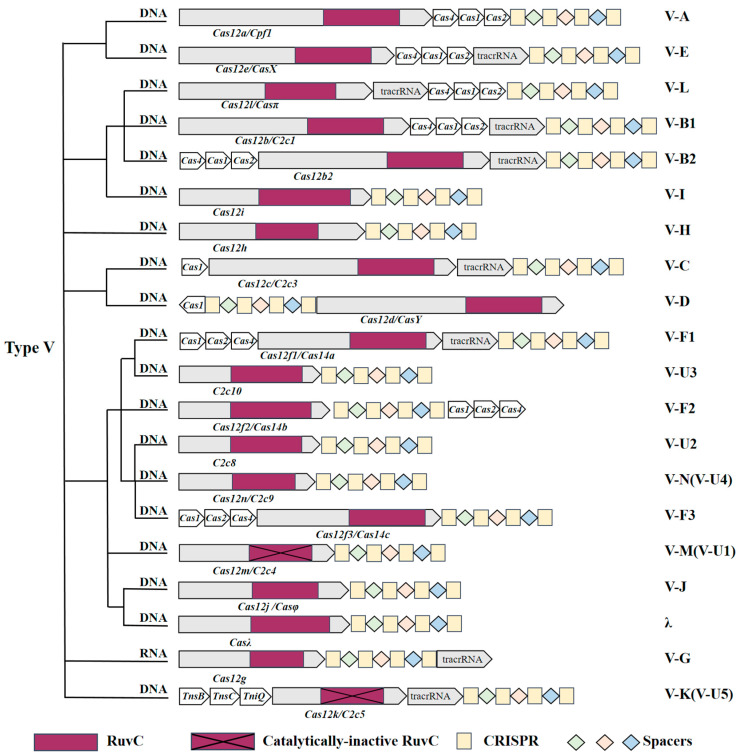
Classification and evolutionary relationships of the type V CRISPR-Cas system. The phylogenetic tree was constructed according to the method of Wu et al. [28], and the additional data of Cas12l, Cas12n and Casλ were incorporated into the phylogenetic tree. Genes or tracrRNAs are shown as pentagons; the yellow rectangle and diamond represent CRISPR and spacers, respectively.

The CRISPR-Cas12 system utilizes a solitary RuvC domain located at the distal PAM end site to facilitate dsDNA cleavage, as well as non-specific cleavage of ssDNA [25]. Multiple CRISPR-Cas12 systems were first identified in 2018 [27]. Subsequently, more and more applications and mechanisms of the new CRISPR-Cas12 system in the genome have been studied [28,29,30]. Up to now, more than 10 subtypes (Cas12a-n) of the type V CRISPR- Cas system have been identified [4,31], including Cas12a, Cas12b, Cas12c, Cas12d, Cas12e, Cas12f, Cas12g, Cas12h, Cas12i, Cas12l (Casπ), Cas12j (CasΦ), and Casλ [14], and five miniature Cas12 variants: V-U1 (Cas12m) [28], V-U2, V-U3 (Cas12f1), V-U4 (Cas12n) [15], and V-U5 (Cas12k) (Figure 2, Table 1).

These Cas12 subtype systems have different functional properties, depending on their structure, mechanism, and evolutionary development. According to the composition of the gRNA structure, some are mediated by a single crRNA. For example, Cas12i [32] does not have tracrRNA but can self-treat pre-crRNA to form mature crRNA and cleave dsDNA targets. Some Cas12 proteins are co-mediated by crRNA and tracrRNA or individually guided by a fused single guide RNA (sgRNA), such as Cas12b and Cas12d.

Most Cas12 proteins have the ability to specifically recognize and cleave dsDNA, including random cleavage of ssDNA or single-stranded RNA (ssRNA) by the activated RuvC domain. For example, Cas12a [33] specifically cleaves dsDNA and non-specifically degrades ssDNA under the guidance of crRNA, and Cas12g [12] specifically recognizes ssRNA substrates and has non-specific cleavage activity after activation.

**Table 1:** Characteristics of the CRISPR-Cas12 system.

Type	Cas12 Protein	Size (aa)	PAM (5′-3′)	Processing precrRNA	tracrRNA	Target	Cleavage Activity	Classic Origin	References
V-A	Cas12a/Cpf1	1223	TTTV	yes	no	dsDNA, ssDNA	cleavage	*Lachnospiraceae bacterium*	[33]
V-B	Cas12b/C2c1	1129	TTN	no	yes	dsDNA, ssDNA	cleavage	*Alicyclobacillus acidoterrestris*	[34]
V-C	Cas12c/C2c3	1218	TN	no	yes	dsDNA, ssDNA	no cleavage	uncultured archaeon	[35]
V-D	Cas12d/CasY	1200	TR	no	scoutRNA	dsDNA	cleavage	hot springs metagenome	[36]
V-E	Cas12e/CasX	986	TTCN	no	yes	dsDNA	cleavage	*Deltaproteobacteria bacterium*	[37]
V-F	Cas12f/Cas14	529	TTN	no	yes	dsDNA, ssDNA	cleavage	uncultured archaeon	[38]
V-G	Cas12g	767	No PAM	no	yes	ssRNA	cleavage	*Acidobacteriota bacterium*	[12]
V-H	Cas12h	871	RTR	yes	no	dsDNA	cleavage	hypersaline lake sedimentmetagenome	[27]
V-I	Cas12i	1093	TTN	yes	no	dsDNA	cleavage	Metagenomic database	[32]
V-J	Cas12j/Casφ	737	TBN	yes	no	dsDNA	cleavage	*Caudoviricetes*	[13]
V-K (V-U5)	Cas12k/C2c5	639	GTN	no	yes	dsDNA	no cleavage	*Scytonema hofmannii*	[39]
V-L	Cas12l/Casπ	860	CCN	no	yes	dsDNA	cleavage	*Armatimonadetes bacterium*	[40]
V-M (V-U1)	Cas12m/C2c4	607	TTN	yes	no	dsDNA	no cleavage	*Gordonia otitidis*	[41]
V-N (V-U4)	Cas12n/C2c9	506	AAN	no	yes	dsDNA	cleavage	*Actinomadura craniellae*	[15]
V-U2	C2c8	-	-	yes	no	dsDNA	cleavage	*Cyanothece* sp. *Pcc sso*	[27]
V-U3	Cas12f1/C2c10	422	TTN	no	yes	dsDNA	cleavage	*Acidibacillus sulfuroxidans*	[42]
-	Casλ	747	TTR	yes	no	dsDNA	cleavage	bacteriophage	[14]

In addition, some Cas12 proteins have no cleavage activity, and they can recognize and bind to target dsDNA or recruit other functional proteins to achieve efficient gene editing at specific gene sites. For example, Cas12c [35] itself has no DNA nuclease activity, but it can cleave pre-crRNA, bind and inhibit gene expression at target DNA sites under the guidance of mature crRNA, and Cas12k [39] and Cas12m [41] mediate RNA-guided Tn7-like transposon translocation.

## 4. Structural Characteristics and Mechanism of the Miniature Cas12 Protein

### 4.1. Cas12f

The Cas12f family proteins are comparatively condensed group Cas proteins (400–700 aa), including Cas12f1 (Cas14a), Cas12f2 (Cas14b), and Cas12f3 (Cas14c) [43]. The Cas12f protein exhibits specific targeting of dsDNA in vitro, and this targeting effect depends on the PAM sequence [11]. The Cas12f protein demonstrates unrestricted targeting of ssDNA and exhibits trans-cleavage activity, independent of the PAM site [44]. The structures of AsCas12f [42], Un1Cas12f [38], and CnCas12f [45] were identified by electron cryo-microscopy (cryo-EM). The results showed that the Cas12f protein has a unique asymmetric dimer configuration, and gRNA guides Cas12f to recognize the PAM sequence and specifically cleave the target sequence.

**Figure 3:**
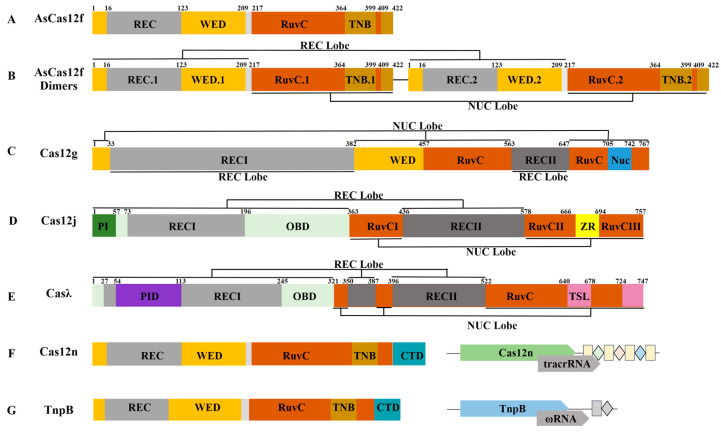
Domain organization of the miniature Cas12 proteins. (**A**–**E**) Diagram of the domain organization of AsCas12f, AsCas12f dimers, Cas12g Cas12j, and Casλ; (**F**,**G**) Diagram of the domain organization and biochemical features of Cas12n and TnpB.

Taking AsCas12f as an example, AsCas12f monomer consists of four parts: Wedge (WED) domain, Recognition (REC) domain, RuvC domain, and Target nucleic acid binding (TNB) domain [46] (Figure 3A). When AsCas12f functions, two AsCas12f monomers (AsCas12f.1 and AsCas12f.2) interact with sgRNA and target sequences to form AsCas12f-sgRNA-DNA complexes in an asymmetric dimer structure [42]. The AsCas12f dimers is a double-leaf structure composed of REC Lobe (Recognition lobe) and NUC Lobe (Nuclease lobe). The sgRNA and target sequence are located in the central channel between the two leaves. REC Lobe is composed of WED and REC domains of AsCas12f.1 and AsCas12f.2 (WED.1/WED.2/REC.1/REC.2), while NUC Lobe is composed of RuvC and TNB domains of AsCas12f.1 and AsCas12f.2 (RuvC.1/RuvC.2/TNB.1/TNB.2) (Figure 3B).

### 4.2. Cas12g

Cas12g, a RNA-guided ribonuclease in the CRISPR-Cas12 system that targets and cleaves ssRNA [47]. Cas12g has a bilobed structure and is composed of REC Lobe and NUC Lobe (Figure 3C). REC Lobe is distributed in a dumbbell shape and consists of the RECI and RECII domains. It is responsible for identifying substrates and folding into inverted ‘F’-shaped sgRNA. NUC Lobe is composed of the WED, RuvC, and NUC domains and is concentrated in three-dimensional space. The sgRNA is located in the central channel between the two leaves of REC Lobe and NUC Lobe, which facilitates the conformational change of the two leaves and activates the Cas12g protein [12].

### 4.3. Cas12j

Cas12j, also known as Casφ, a class of RNA-guided micronucleases, which is found in phages and containing 700–800 aa [13]. Cas12j is composed of REC Lobe and NUC Lobe (Figure 3D), which is a bilobed structure. REC Lobe includes two REC (REC I and REC II), OBD (Oligonucleotide-binding domain), and PAM-interacting (PI) domains, which are responsible for recognizing PAM sequences, promoting dsDNA unwinding, and guiding crRNA to bind to target DNA strands to form a R-ring structure. NUC Lobe consists of zinc ribbon (ZR) and RuvC (RuvCI, RuvCII, and RuvCIII) domains. The ZR domain helps to recruit DNA substrates to the active region of RuvC. RuvC processes crRNA to cut dsDNA, by specifically recognizing the 5′-TBN-3′ PAM sequence near the crRNA complementary DNA sequence through its active site [48,49]. Cas12j unwinds dsDNA by recognizing the PAM site and catalyzes the formation of the R-ring. Its spatial state is changed from a typical double-leaf structure to a T-shaped structure, which facilitates the sequential cleavage of dsDNA. Cas12j can specifically cleave dsDNA and can collateral ssDNA. Cas12j non-specifically cleaved ssDNA after the cleavage of DNA [50,51].

### 4.4. Casλ

Casλ is a novel Cas protein found in phages with the ability to self-treat the precursor CRISPR RNA (pre-crRNA) [52]. At present, it contains 55 miniaturized proteins in the Casλ family, which is the closest relationship with Cas14j. The arrangement of the protein domains is significantly different from the known Cas12 system, and the Casλ is bilobed structure, which is composed of REC Lobe and NUC Lobe, which include OBD, REC (RECI and RECII), PAM-interacting domain (PID), RuvC, and Target Strand Loading (TSL) domains, respectively (Figure 3E). TSL has the function of binding to ssDNA substrates, and its position is similar to the NUC domain. The CRISPR-Casλ protein promotes the target dsDNA unwinding together with the OBD, REC I, and PID domains. The RuvC domain processes the pre-crRNA and obtains the mature crRNA. The crRNA will adopt a slender hairpin structure, thereby acquiring the ability to perform cis-cleavage. Additionally, Casλ exhibits trans-cleaving activity towards both ssDNA and ssRNA subsequent to the recognition and cleavage of dsDNA [14,30,53].

### 4.5. Cas12n

Cas12n, a V-U4-type Cas12 protein, is recently discovered and known as C2c9, which has only 400–800 aa and recognizes a rare 5′-AAN-3′ PAM sequence. Cas12n is a monomeric protein that is composed of WED, REC, RuvC, and C-terminal domain (CTD) and encodes tracrRNA. Cas12n, crRNA, and tracrRNA can form complexes to help Cas12n recognize PAM sequences, target dsDNA, and produce two cis-cleavages to form staggered dsDNA. Cas12n also has the ability to cut trans. Cas12n and TnpB transposon-related ribonucleoproteins (the ancestor of the type V CRISPR-Cas12 system protein) have high similarity in gRNA source, protein structure, and sequence arrangement (Figure 3F,G). Phylogenetic tree analysis showed that the two had the closest genetic relationship. Therefore, Cas12n may be the intermediate product of TnpB evolving into other type V Cas12 nucleases [15,54].

## 5. Application of the Miniature CRISPR-Cas12 System

**Figure 4:**
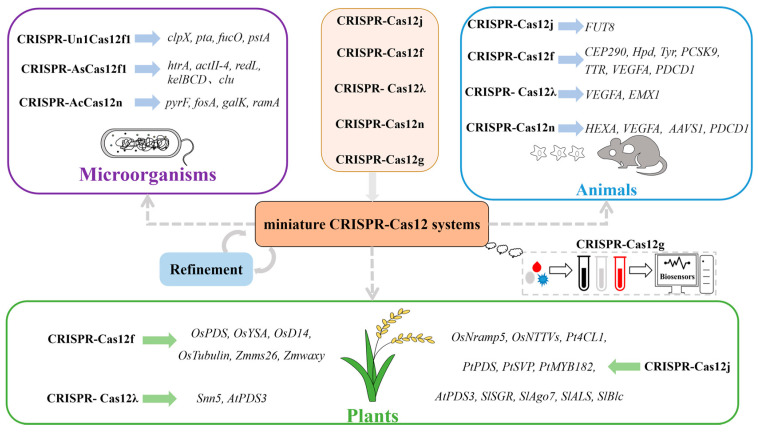
Genome editing with the miniature CRISPR-Cas12 system.

Recently, the miniature Cas12 protein has set off a new wave of gene editing revolution due to its small molecular weight, low off-target efficiency, high editing activity, and high selectivity of editing sites. The powerful genome editing ability of the Cas12 protein has been verified in microorganisms, animals, and plants (Figure 4, Table 2).

### 5.1. Genome Editing of Microorganisms

Microorganisms play important roles in the field of life sciences, which have been widely used in many industries such as pharmaceuticals, biotechnology, and cosmetics manufacturing through by-products of metabolic processes, such as enzymes and antibiotics [65]. However, despite the immense potential of microorganisms in various aspects of human life, our current utilization falls short of its full capacity. The optimization of microorganism characteristics through genome editing and modification techniques enables us to better address specific needs, thereby enhancing their applicability across diverse fields. The miniature CRISPR-Cas12f can cleave dsDNA in vitro by relying on PAM sequences rich in 5′-T or 5′-C in bacteria [66]. Compared to CRISPR-SpCas9 and CRISPR-Cas12a, the miniature CRISPR-Cas12f system has less volume and cytotoxicity and is more suitable for editing some industrial-related bacteria. The editing efficiency of Un1Cas12f1 to edit four non-essential genes in *Escherichia coli* strains MG1655 and BW25113 was 63–100%, which was much higher than that of the CRISPR-SpCas9 system of 50–79% [55]. The CRISPR-AsCas12f1 genome editing system efficiently and accurately deleted large fragments in the *hisD* gene and *cyaA* gene of *E.coli*. The edited *E. coli* produced a phenotype that could not perform histidine de novo biosynthesis and slow growth under aerobic conditions. The system can also accurately edit genes related to metabolism and drug resistance of *Klebsiella pneumoniae* [43]. In addition, when editing the *lef* gene in the pXO1 plasmid, the *htrA* gene of *Bacillus anthracis,* and the single gene of *Streptomyces hygroscopicus* M145, the CRISPR-AsCas12f1 system showed 100% editing efficiency [56,57]. When editing double genes (*actII-4* + *redL*) and double gene clusters (*act* + *red*), the editing efficiency of the CRISPR-AsCas12f1 system can reach 46.7% and 40% [57].

In addition, CRISPR-AsCas12f1 has also been proven to be a low-toxic and efficient genome editing tool and has been successfully applied to the gene editing of *Streptomyces hygroscopicus* SIPI-054, an industrial producer of Milbemycin, to efficiently delete the DNA regions of the *kelBCD* gene and *clu* gene [57]. CRISPR-AcCas12n successfully targeted the *pyrF*, *fosA*, *galK,* and *ramA* genes of *Klebsiella pneumoniae*, and the editing efficiencies were 99.97%, 99.84%, 99.71%, and 99.85%, respectively [15,54]. These results fully demonstrated the reliability and effectiveness of the miniature CRISPR-Cas12 system in bacterial genome editing.

### 5.2. Genome Editing of Humans and Animals

In the field of gene editing, adeno-associated virus (AAV) and lipid nanoparticles (LNP) are the most commonly used delivery systems in animal gene editing, but the traditional CRISPR-Cas system is bulky and difficult to deliver, which makes it easy to cause an immune response [67,68,69,70]. Recently, several research teams have developed a variety of efficient micro-gene editing systems in mammalian cells through bioinformatics big data mining, high-throughput screening, gRNA- and protein-directed modification, Primer-extension-mediated Sequencing (PEM-seq) off-target analysis technology, and in vitro and in vivo gene therapy effectiveness analysis [11,71]. For example, through electroporation and AAV delivery, Un1Cas12f1 [58], Cas12j (CasΦ-2 and Cas12j-SF05) [13,51], AcCas12n [15], and Casλ [14] have successfully achieved efficient and specific genome editing in HEK293 and HEK293T cells.

Using AAV delivery and embryo injection of Un1Cas12f1 mRNA and gRNA, *Hpd* and *Tyr* genes were successfully targeted, resulting in gene-edited mice with gene editing efficiency of about 20% [59,72]. Two miniature CRISPR-Cas12f systems: enOsCas12f1 (enhanced OsCas12f1, 433 aa, 5′-TTN) and enRhCas12f1 (enhanced RhCas12f1, 415 aa, 5′-CCD) have low off-target, high efficiency, and a wide targeting range in mammalian cells. Based on enOsCas12f1 and enRhCas12f1, researchers have developed a chemical small molecule Trimethoprim-induced DD-enOsCas12f1 system by integrating destabilized domain (DD) and enOsCas12f1 [60]. Through AAV delivery, exon51 can be edited in Duchenne muscular dystrophy (DMD) model mice, and the expression of dystrophin can be effectively restored [60]. Based on the enOsCas12f1 mutants (dead enOsCas12f1 and denOsCas12f1), researchers have further developed an epigenetic editing system mini-CRISPR-off and a gene expression activation system denOsCas12f1-VPR, which can mediate efficient DSB-free gene expression regulation in mammalian cells [60].

In addition, the natural CRISPR-AsCas12f1 system was effectively transduced into three human cells (HEK293, U-2 OS, and Huh-7) by a single AAV vector and successfully edited *VEGFA* and *PDCD1* genes [43]. In induced pluripotent stem cells (iPSCs) of DMD patients, the AsCas12f-HKRA mutant can effectively restore the expression of dystrophin and regulate the level of plasma transthyretin (TTR) [42]. In in vivo experiments in mice, AsCas12f-HKRA can target the *TTR* gene for transthyretin amyloidosis, effectively reduce the expression of TTR, and can also knock in *EGFP* and coagulation factor *F9* genes by HDR to achieve gene therapy for hemophilia mouse models [42]. Cas12j-8 and Cas12j-10 are newly established CRISPR-Cas systems, which have efficient and specific editing activity and can distinguish single base differences when targeting single nucleotide polymorphism (SNP) sites in mammalian cells [73]. In summary, the development of the micro Cas12 system and its derivatives provides a powerful new tool for precision medicine and gene therapy.

### 5.3. Genome Editing of Plants

Gene editing tools play an important role in the field of plant research, which not only promotes the study of plant gene function but also provides an effective technical means for crop genetic improvement [74,75,76]. Using the miniature CRISPR-Cas12 system, the genomes of wheat, maize, rice, and other crops can be accurately edited to create excellent traits, increase crop yield, and enhance resistance to pests and diseases.

In *Arabidopsis thaliana*, Cas12j can be used to edit the *AtPDS3* gene in *Arabidopsis* protoplasts and can also produce stable genetic gene editing mutants in *Arabidopsis* thaliana, but the overall editing efficiency is not high (<1%) [13]. The optimized vCas12j and nCas12j can show higher editing efficiency (6.12% and 6.07%) [13,61,77]. Studies have confirmed that the editing efficiency of Casλ on the endogenous *AtPDS3* gene can reach 18%, which is significantly higher than the editing efficiency when Cas12j was used previously. In addition, the researchers also used Casλ to edit the endogenous resistance *Snn5* gene in hexaploid wheat protoplasts [14]. In rice protoplasts, Cas12j-mediated multi-gene editing sites achieved 15–50% efficient editing, but in rice and poplar stably inherited plants, the mutation efficiency was very low, and the editing efficiency was only 1.5–20%. The editing efficiency of Cas12j-SF05 in rice endogenous gene *OsNramp5* was only 20% [51,62].

Cas12f is also a powerful plant genome editing tool. SpCas12f was infected with rice callus by Agrobacterium tumefaciens, and heritable mutations were successfully introduced at multiple target sites. The mutation efficiency was 28.8% and 55.6%, respectively. The main mutation was deletion mutation, and no off-target mutation of SpCas12f was found [64]. AsCas12f-HKRA also had editing activity in the editing test targets of three rice genes: *OsPDS*, *OsYSA,* and *OsD14*, and the editing efficiency, respectively, reached 27.1%, 24%, and 53.1% [63]. In addition, miniature CRISPR-Cas12 can also be used for genome editing in many plant species, such as tobacco, tomato, maize, etc. [77,78].

The miniature Cas12 system not only has the ability of DNA site-directed deletion but also can be coupled with other functional proteins for base editing, transcriptional activation, and epigenetic modification. For example, by constructing the inactive protein dCasΦ2, the dCasΦ2-cytosine base editor (dCasΦ2-CBE) and the dCasΦ2-adenine base editor (dCasΦ2-ABE) were developed, which achieved efficient and specific single base editing in wheat and rye. The editing ranges of C-to-T and A-to-G are C2 to C17 (the second cytosine base to the 17th cytosine base of the target sequence) and A9 to A11, respectively [79].

The transcriptional activation system was constructed by combining Cas12j2 with the methylase catalytic domain, and the transcriptional activation of endogenous genes *OsER1* and *OsNRT1.1A* was realized in transient transformation of rice protoplasts and stable genetic transformation of regenerated plants. Cas12j2 can also be coupled with the methylase catalytic domain to form a new plant genomic DNA methylation epigenetic editing system. By epigenetic editing the promoter region of rice endogenous gene *OsGBSS1*, the level of cytosine methylation modification was significantly increased, thus effectively inhibiting the transcription level of *OsGBSS1* [62,80].

## 6. Common Strategies to Improve the Editing Efficiency of Miniature CRISPR-Cas12 Systems

The miniature CRISPR-Cas12 system has attracted much attention due to its high specificity and small size, which has brought about revolutionary progress in many fields, such as synthetic biology gene therapy and crop genetic improvement. However, it is also found in the study that the natural miniature CRISPR-Cas12 system generally has the problem of low editing efficiency in vivo. Therefore, the continuous development of an accurate and efficient miniature CRISPR-Cas12 system is still a research hotspot in the field of life science at present and in the future (Table 3).

### 6.1. Optimization of gRNA Design

gRNA is the “navigation system” of CRISPR-Cas and guides the Cas protein to accurately locate and cut the target DNA sequence to achieve the aim of gene editing. Chemical modification of gRNA ends and stem-loop structures in gRNAs are two ways to optimize the gene editing efficiency [81,82].

**Figure 5:**
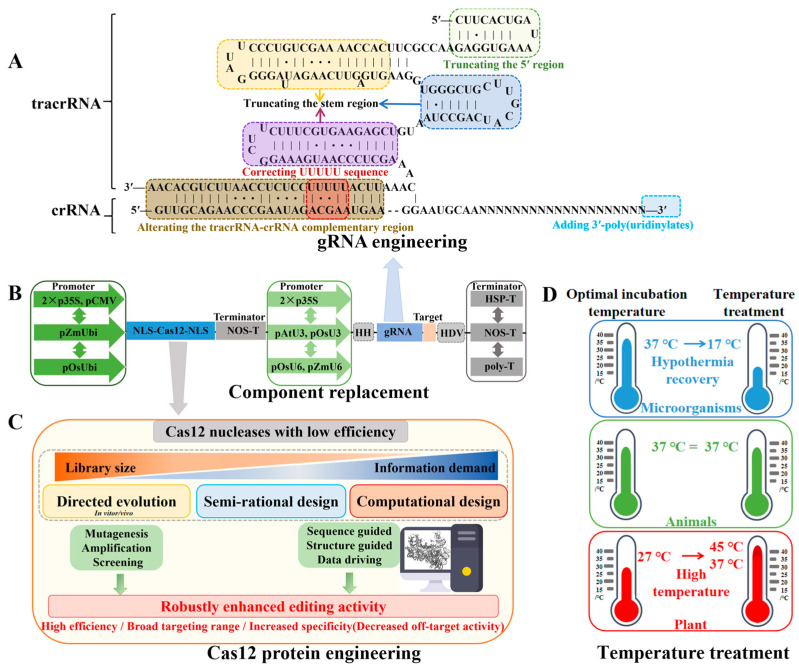
Common strategies to improve the editing efficiency of miniature CRISPR-Cas12 systems. (**A**) Schematic diagram of the gRNA structure optimization site of Un1Cas12f1. The boxes of different colors in the figure represent the 5′ end of tracrRNA, three stem-loop sequences of tracrRNA, the tracrRNA-crRNA complementary region, the five uridine monophosphate internal sequences on tracrRNA, and the 3′ end of crRNA, respectively. (**B**) Optimized schematics of different action elements. The arrows represent promoter elements; the dark gray rectangles represent terminator elements. (**C**) Cas12 protein optimization diagram. (**D**) The optimization diagram of temperature treatment to improve the editing efficiency of Cas12. Blue, green, and red represent the culture temperatures of different species, respectively.

For example, five modification sites (MS) were optimized on natural tracrRNA and crRNA sequences, including five uridine monophosphate internal sequences on tracrRNA, the 3′ end of crRNA, the 5′ end of tracrRNA, the tracrRNA-crRNA complementary region, and the stem-loop sequence of tracrRNA, respectively. These five optimization sites (M1-M5) can greatly improve the editing efficiency. The co-optimized Un1Cas12f1_ge4.1 system is 867-fold higher than the original Un1Cas12f1 system on average and can achieve efficient and specific genome editing through AAV delivery, showing the same editing ability as SpCas9 [58] (Figure 5A).

CWCas12f (derived from *Candidatus Woesearchaeota archaeon*) and Un1Cas12f1 have almost the same gene sequence. CWCas12f using natural gRNA also did not show significant editing activity. The optimized gRNA-TaRGET-guided CWCas12f can greatly improve its gene editing efficiency (20%) and accuracy [83]. The tracrRNA of SpaCas12f1 has a unique head-to-toe hairpin structure, which is the main factor limiting its effective editing in mammalian cells. Based on gRNA-MS1, the research team designed 18 different modification sites (MS2–MS19), and finally obtained a highly efficient editing gRNA: gRNA_MS13, which made the editing efficiency more than two-fold higher than MS1, thus transforming CRISPR-SpaCas12f1 into a highly efficient genome editor comparable to CRISPR-FnCas12a [84]. By optimizing gRNA, it was found that Al2Cas12f1-tracrRNA-tR3 is the best configuration for human cell editing [85] and can also transform the originally ineffective CRISPR-EsCas12f1 system into an efficient bacterial genome editor [86].

CRISPR-Cas12n is a kind of miniature gene editing system that can recognize the rare AAN PAM sequence. TracrRNA is located inside of the Cas12n mRNA and an efficient optimized version of the AcCas12n system. Cas12 Pro is designed by using the sgRNA system. It has up to 80% editing efficiency in human cells and can perform base editing [15]. The function of Cas12j has been widely reported in plants, and the gene editing efficiency in *Arabidopsis* protoplasts is less than 1% [13]. By optimizing the stem-loop structure of crRNA, the most efficient and conservative stem-loop variant of Cas12j2 crRNA-TACG was screened, which increased the overall efficiency of Cas12j2 by two-fold. Subsequently, based on the efficient crRNA-TACG stem-loop variant and the optimal spacer length (18nt), the Cas12j2 protein and its corresponding crRNA were coordinated and optimized by a single transcript unit (STU), and an efficient optimized version Cas12j2-STU.v2 was created. In the single-target editing and multi-target editing regenerated rice plants, the efficiency was as high as 78.8%, which increased by about five-fold [87].

**Table 3:** Strategies for improved genome editing.

Optimization Method	Gene Editing System	Processing	Efficiency	Activity	Species	References
gRNA engineering	Un1Cas12f1_ge4.1	ge4.1 (MS2, MS3, MS4, MS5)	867-fold	<1%→15.2% ± 12.0%	HEK293T	[58]
	SpaCas12f1-gRNA_MS13	MS13 (M10, AAGG, MS8)	Improvement	<1%→<21.95%	HEK293T	[84]
	CWCas12f1-TaRGET	TaRGET	20-fold	<1%→20%	HEK293T	[83]
	Al2Cas12f1-tR3	tR1-Stem1 truncation	16-fold	0.5%→8.2%	HEK293T	[85]
	Cas12Pro	sgRNA_v6	2-fold	80%	HEK293T	[15]
	Cas12j2-TACG	crRNA-TACG	2-fold	~40%	*Oryza sativa*	[87]
	Cas12j2-STU.v2	STU + crRNA-TACG	5-fold	78.80%	*Oryza sativa*	[87]
Cas protein engineering	nCasΦ, vCasΦ	E159A, S160A, S164A, D167A, E168A, Δ155–176 (GSSG)	20-fold	-	PCR product	[48]
	nCas12j2	E159A, S160A, S164A, D167A, E168A	Improvement	50.00%	*Oryza sativa*	[62]
	Cas12j2-STU.v4-S511	Npu split-S511	Improvement	6.5%→7.1%	*Oryza sativa*	[87]
	enRhCas12f1	L270R, Rh-sg1.1	1.7-fold	23.3 ± 26.8%	HEK293T	[60]
	enOsCas12f1	T132R, D52R Os-sg2.6	3.9-fold	54.7 ± 29.8%	HEK293T	[60]
	AsCas12f1-v5.1	N70Q, K103R, A104R, S118A, D364R, sgRNA_T1	1.5–13.5-fold	-	HEK293T	[88]
	enAsCas12f	D196K, N199K, G276R, N328G, D364R, sgRNA-v2	11.3-fold	69.80%	HEK293T	[46]
	AsCas12f-YHAM	F48Y, S188H, V232A, E316M, sgRNA_ΔS3-5_v7	12.6-fold	3.0%→40.8%	HEK293T	[42]
	AsCas12f-HKRA	I123H, D195K, D208R, V232A, sgRNA_ΔS3-5_v7	13.9-fold	3.0%→44.7%	HEK293T	[42]
	AsCas12f-YHAM	F48Y, S188H, V232A, E316M, sgRNA_ΔS3-5_v7	Improvement	4.0%, 0%, 25.0%	*Oryza sativa* (*OsPDS*, *OsYSA*, *OsD14*)	[63]
	AsCas12f-HKRA	I123H, D195K, D208R, V232A, sgRNA_ΔS3-5_v7	Improvement	27%, 24%, 53%	*Oryza sativa* (*OsPDS*, *OsYSA*, *OsD14*)	[63]
Temperaturre treatment	Casλ	23 °C, 28 °C, 32 °C	Improvement	0%, ~2%, ~15%	*Arabidopsis thaliana*, *Triticum aestivum*	[14]
	SpCas12f	45 °C	Improvement	20–50%, 1–1.5%	*Zea mays* L. (*Zmms26*, *Zmwaxy*)	[89]
	SpCas12f	30 °C	Improvement	28.8%, 55.6%	*Oryza sativa* (Tub-1, Tub-2)	[64]
	AsCas12f1	42, 27, 17 °C	Improvement	1%, 46%, 86%	*Escherichia coli* (Double Target)	[90]
	AsCas12f1	37, 17 °C	Improvement	3%, 35%	*Escherichia coli* (Triple Target)	[90]
Other	Un1Cas12f1	pOsU3	3-fold	0.16–1.14%→0.34–3.70%	*Oryza sativa*	[78]
	Cas12j2	pAtU3	Improvement	0.29–2.66%	*Solanum lycopersicum* L.	[13]
	Cas12j2	HH-HDV, Pol II	Improvement	0–40%→15–50%	*Arabidopsis thaliana*, *Oryza sativa*	[62]
	eCWCas12f-VPR	ge4.1; D171R, T175R, E179A; D171R, T175R, K358R, E556R; NLS; FUS-IDR	32.48-fold	-	HEK293T	[91]
	miniCRa	sgRNA (20nt), D143R, T147R, T203R	4-fold	-	HEK293T, U2OS	[92]
	SminiCRa (Sso7d-dUn1Cas12f1-3R-VPR-C)	Sso7d, miniCRa	29.65-fold	-	HEK293T, U2OS	[92]
	miniCRi	dUn1Cas12f1-3R-KRAB	improvement	-	HEK293T	[92]
	SminiCRi (Sso7d-dUn1Cas12f1-3R-KRAB)	Sso7d, miniCRi	improvement	-	HEK293T, Huh-7	[92]
	STUminiABE	Sso7d, D143R, T147R, K330R, E528R	4.4-fold	<10%→54%	HEK293T	[93]
	STUminiCBE	Sso7d	3.5-fold	<10%→45%	HEK293T, Huh-7	[93]

### 6.2. Cas Protein Engineering

In the CRISPR-Cas9 system, the editing efficiency and specificity of the Cas9 protein can be improved by the strategies of directed evolution, crystal electron microscopy structure analysis, and deep mutation scanning [94,95,96,97]. Recently, cryo-EM was used to analyze the function of a large number of miniature Cas12 proteins, and it was found that the cleavage activity of the natural Cas12 protein was moderate. The editing efficiency of the miniature CRISPR-Cas12 system was also improved by the analysis of the protein crystal structure, directed evolution, or combination with gRNA engineering (Figure 5C).

The conformational changes of CRISPR-CasΦ (Cas12j) in recognizing DNA, activating nuclease, and cleaving DNA were found by cryo-EM. By comparing the state of Cas12j before and after binding to DNA, it was revealed that Cas12j rearranged the sites for DNA cleavage when recognizing DNA. Based on these structures, multiple alanines were introduced to replace the original amino acid sequence, and two mutants, nCasΦ (E159A, S160A, S164A, D167A, and E168A) and vCasΦ (Δ155–176 (GSSG)), were successfully obtained. The results showed that the cleavage speed of these mutants was 20-fold faster than that of natural Cas12j [48]. In rice protoplasts, Cas12j2, vCas12j2, and nCas12j2 have editing activity for 15 rice endogenous genes. The editing activity of the vCas12j2 variant is comparable to that of nature Cas12j2, while the activity of the nCas12j2 variant at multiple sites is significantly higher than that of the Cas12j2 and vCas12j2 variants [62].

In addition, through protein structure analysis, a cleavage site was introduced at the RecII domain of the Cas12j2-STU.v4 protein. The split Cas12j2-STU.v4-S511 was cleaved by intein in rice protoplasts, and the editing efficiency of Cas12j2-STU.v4-S511 was consistent with that of Cas12j2. This finding provides data support for the viral vector delivery CRISPR-Cas12j system [87]. By replacing the amino acid in the REC/RuvC domain of RhCas12f1 and OsCas12f1 with arginine, two CRISPR-Cas12f1 systems with stronger editing activity and wider targeting range were found: enOsCas12f1 (T132R and D52R) and enRhCas12f1 (L270R) [60].

An enhanced version of the genome editor AsCas12f1-v5.1 (N70Q, K103R, A104R, S118A, D364R, and sgRNA_T1) was obtained by optimizing the gRNA corresponding to AsCas12f1, and its activity in animal genome editing was found to be greatly enhanced [88]. Another variant, enAsCas12f (D196K, N199K, G276R, N328G, D364R, and sgRNA-v2), can achieve up to 69.8% insertion and deletion of DNA fragments at specific genomic sites, while showing low off-target editing activity [46].

More reliable and efficient AsCas12f-YHAM and AsCas12f-HKRA are developed by deep mutational scanning (DMS) and structural enhancement design, which significantly improved their genome editing activity on multiple target sequences of human cell lines, with an average editing efficiency of 40.8% and 44.7%, respectively [42]. In rice, the editing efficiency of AsCas12f-YHAM in the *OsPDS* and *OsD14* genes reached 4% and 25%, respectively, while no editing results were observed in *OsYSA*; the editing efficiency of AsCas12f-HKRA in these three genes reached 27.1%, 24%, and 53.1%, respectively. This indicates that AsCas12f-HKRA shows better substrate sequence compatibility and higher editing efficiency on these test targets [63].

### 6.3. Selection of Treatment Temperatures

The optimal temperature for the enzyme activity of the Cas9/Cas12a protein is 37 °C, but 27 °C is the most suitable temperature for most plant tissue cultures [98,99]. The editing efficiency of five different Cas endonucleases: SpCas9, SaCas9, St1Cas9, Mb3Cas12a, and AsCas12a in rice callus is detected at 37 °C and 27 °C (Figure 5D). At the optimal temperature of 37 °C, five different Cas9 and Cas12a protein endonucleases can expand the editing range and efficiency of the target sequence regardless of monoallelic editing or biallelic editing [100].

The temperature has a greater impact on the activity of the miniature Cas12 protein. The optimal reaction temperature for SpCas12f1 and AsCas12f1 nuclease to bind and cleave dsDNA is 45–55 °C. After heat shock treatment at 45 °C for 4 h per day for 3 days, SpCas12f is successfully introduced into two agronomic-related genes (*Zmms26* and *Zmwaxy*) in maize protoplasts and stable expression lines [89]. After tissue culture at 30 °C under constant light, rice calli edited by SpCas12f grows rapidly and vigorously and has high mutation frequencies at the Tub-1 and Tub-2 sites, which were 28.8% and 55.6%, respectively [64].

Casλ is also a temperature-dependent protein. In *Arabidopsis* and wheat protoplasts, no site-directed mutagenesis occurred under 23 °C culture conditions. With the increase in culture temperature, genome editing began to be induced at 28 °C. When the temperature increased to 32 °C, the genome editing efficiency reached the highest [14]. CRISPR-AsCas12f1 is used for multiple genome editing at the single nucleotide level in *E. coli*. By allowing cells to recover at a lower temperature during the editing process, they found that this treatment could significantly improve the editing efficiency of editing two different targets simultaneously [90]. These studies have highlighted the potential of temperature optimization in improving genome editing efficiency.

### 6.4. Other Optimization Methods

In addition to optimizing gRNA design and Cas protein engineering and selecting the optimal temperature treatment strategy, the editing efficiency of the miniature CRISPR-Cas system can be improved by optimizing the driving elements, adding nuclear localization signal (NLS), and innovating delivery methods (Figure 5B).

When RNA polymerase II (Pol II) and RNA polymerase III (Pol III) promoters were used to drive Un1Cas12f1-ge4.1, the editing efficiency of Pol III promoter (pOsU3-ge4.1)-driven gRNA was three-fold higher than that of Pol ll (pZmUbi1-ge4.1), and the editing efficiency was 0.34–3.70% and 0.16–1.14%, respectively [78]. In tomato protoplasts, the editing efficiency of CRISPR-Cas12j using the Pol III (pAtU3) promoter was also better than that of the Pol II (2 × p35S) promoter. The CRISPR-Cas12j2 system was initially edited in *Arabidopsis* with an efficiency of only about 1% [13]. In rice protoplasts, half of the 34 target sites have no editing phenomenon, and the editing efficiency of the other half of the target sites was 0–40%. The CRISPR-Cas12j2 system increases the editing activity in rice protoplasts to 15–50% based on HH-HDV elements and dual polymerase II (Pol II) promoters [62]. In summary, through the application of Cas12f, Cas12j and Casλ in plant genome editing, it has been observed that the wild-type miniature Cas12 protein does not have efficient editing ability. It is a practical optimization strategy to drive the miniature CRISPR-Cas12 system by optimizing the structure of gRNA and Cas protein and selecting promoters adapted to the corresponding plants. In addition, the addition of some auxiliary elements can also help the miniature Cas12 protein to improve the editing efficiency and achieve accurate editing.

Miniature Cas12f effector protein mutants can fuse transcriptional regulators to perform accurate and specific gene activation or gene inhibition in cells. The transcriptional activation system dCasMINI-VPR achieves efficient activation of disease-related genes and has similar specificity to dCas12a-VPR by binding the transcriptional activator Viral Protein R (VPR) to dCasMINI. However, the efficiency of transcriptional regulation varies among different genes, and it is necessary to further optimize the gene expression regulation system [58,101].

The powerful transcriptional activation system miniCRa can activate 1319-fold expression of the *ASCL1* gene by optimizing Un1Cas12f1 and its corresponding sgRNA. Subsequently, the non-specific DNA binding protein Sso7d was combined with miniCRa to develop an enhanced transcriptional activation system, SminiCRa, which further enhanced the binding ability of Un1Cas12f1-3R to DNA, reaching 5628-fold *ASCL1* gene activation, and the transcriptional activation ability of SminiCRa in various lineage cells is broad-spectrum. The optimized Un1Cas12f1 system can also combine the transcriptional repressor with Sso7d for transcriptional inhibition. The results showed that miniCRi (dUn1Cas12f1-3R-KRAB) and SminiCRi (Sso7d-dUn1Cas12f1-3R-KRAB) can mediate an average of about 80% inhibition of multiple test genes, but there is no significant difference in inhibition efficiency between them [92]. Sso7d can also promote efficient base editing. After fusing Un1Cas12f1 with Sso7d, an efficient base editor, STUminiBEs, was developed using truncated sgRNA. Among them, STUminiABE showed an average efficiency of 54% in the conversion from A to G, and STUminiCBE showed an average efficiency of 45% in the conversion from C to T [93].

In addition, the position and combination of the linker peptide and NLS are also very important. A specific and highly expressed regulatory system, eCWCas12f-VPR, was developed by optimizing CWCas12f and linking transcriptional activators using a combination of different lengths of linkers and NLS. Compared to the LbCas12a system, the activation efficiency of eCas12f-VPR on different human genes was increased by 32.48-fold, and the specificity was increased by 26.06-fold [91].

## 7. Conclusions and Perspectives

The discovery of the CRISPR-Cas system has revolutionized gene editing technology, ushing in a new era across various domains, including gene therapy, agricultural biotechnology, and fundamental biological research. Among them, the CRISPR-Cas9 and CRISPR-Cas12 systems are widely used, which can skillfully and conveniently complete genome editing in microorganisms, animals, and plants. However, with the deepening of research, their defects and limitations have been exposed, such as off-target problems, single PAM sites, low editing efficiency of specific sites, and delivery difficulties due to excessive volume [102].

Recently, several miniature Cas12 systems with cleavage activity have been continuously reported due to their small size (400–800 aa), low off-target efficiency, and abundant PAM sites. Their powerful genome editing capabilities (such as insertion and deletion, base substitution, and gene activation) have set off a new round of gene editing revolution [103]. Cas12g is the only Cas12 system with RNA targeting ability [104], which can perform cis-cutting and trans-cutting at the target site. The small volume makes it very suitable for insertion into viral vectors such as AAV to treat genetic diseases and infectious diseases caused by RNA molecular mutations. Cas12f and Cas12n are gRNA-mediated micronucleases, and they differ in structural and functional characteristics [54]. Similar to Cas12a, both Cas12j and Casλ can process their own crRNAs and use non-classical crRNAs for efficient genome editing. They are very similar in structure, function, mechanism of action, and evolutionary origin. These miniature Cas12 systems can directly ignore the packaging limitations encountered in viral vectors and exhibit efficient editing capabilities in microorganisms and animal cells. In plant gene editing, the editing ability of the miniature Cas12 system is generally low, which may be due to the large difference between the optimal activity temperature of the type V system and the optimal temperature of plant tissue culture [105]. It is also necessary to further optimize the gRNA and Cas protein or perform temperature treatment and adjustment of action elements by analyzing their structural characteristics and mechanism of action, thereby greatly improving the editing efficiency of the micro-Cas12 system in plant gene editing [106,107].

The small size and efficient cleavage endow the miniature CRISPR-Cas12 system with unlimited potential. In addition to gene editing in organisms (including base editing, lead editing, transcriptional activation/inhibition, and epigenetic editing), the miniature Cas12 system is also widely used in the rapid diagnosis and detection of various new diseases and the real-time management of plant diseases [108,109,110]. New delivery methods such as viruses and supramolecular nanoparticles (LSNPs) will also become a reality [111,112,113]. Compared to Cas9 and Cas12a nucleases, miniature Cas12 nucleases reduce the frequency of cytotoxicity, off-target effects, large fragment deletions, chromosomal translocations, and random plasmid insertions [11] and also expand the application in animals, plants, and microorganisms. However, most of the current research on the compact type V family is centered on Cas12f and Cas12j, and the discovery of these ultra-miniature gene editing tools is only a beginning. Cas12n, Casλ, and Cas12g are also powerful gene editing tools, which contain a large number of novel functional mechanisms and are expected to introduce many valuable editing tools in the fields of synthetic biology, precision agriculture, gene therapy, and biological sensors. In general, through directed evolution, delivery optimization, temperature treatment, and other means, the development of a miniature Cas12 system in various fields in the future will achieve safer, wider spectrum, and more efficient genome editing.

## Figures and Tables

**Table 2:** Application of the miniature CRISPR-Cas12 system in microorganisms, animals, and plants.

Biological	Miniature Cas12 System	Gene	Efficiency	Species	References
Microorganisms	Un1Cas12f1	*clpX*, *pta*, *fucO*, *pstA*	63–100%	*Escherichia coli*	[55]
	AsCas12f1	*htrA*	100.00%	*Bacillus anthracis*	[56]
	AsCas12f1	(*actII*-*4* + *redL*), (*act* + *red*)	46.7%, 40%	*Streptomyces hygroscopicus* M145	[57]
	AsCas12f1	*kelBCD*, *clu*	70%, 30%	*Streptomyces hygroscopicus* SIPI-054	[57]
	AcCas12n	*pyrF*, *fosA*, *galK*, *ramA*	99.97%, 99.84%, 99.71%, 99.85%	*Klebsiella pneumoniae*	[15]
Animals	Un1Cas12f1	*CEP290*	46.00%	HEK293T	[58]
	Un1Cas12f1	*Hpd*, *Tyr*	20.00%	mouse zygotic embryos	[59]
	enRhCas12f1	*PCSK9*, *TTR*, *VEGFA*	23.3 ± 26.8%	HEK293T	[60]
	enOsCas12f1	*PCSK9*, *TTR*, *VEGFA*	54.7 ± 29.8%	HEK293T	[60]
	enOsCas12f1	exon51	22.7 ± 9.2%	whole muscle	[60]
	AsCas12f1	*VEGFA*, *PDCD1*	11.50%	HEK293, U-2 OS, and Huh-7	[43]
	AsCas12f-HKRA	*TTR*	66.30%	mouse liver	[42]
	CasΦ−2 (Cas12j)	*EGFP*	33%	HEK293	[13]
	Cas12j-SF05	*FUT8*	~12%	CHO cell	[51]
	AcCas12n	35 different human genomic sites	2.2–73.7%	HEK293T	[15]
	Casλ	*VEGFA*, *EMX1*	20–50%, 1–1.5%	HEK293T	[14]
Plants	CasΦ−2 (Cas12j)	*AtPDS3*	0.85%	*Arabidopsis thaliana*	[61]
	vCas12j, nCas12j	*AtPDS3*	6.12%, 6.07%	*Arabidopsis thaliana*	[61]
	Casλ	*AtPDS3*	18%	*Arabidopsis thaliana*	[14]
	Casλ	*Snn5*	<4%	*Triticum aestivum* L.	[14]
	Cas12j-SF05	*OsNramp5*	20%	*Oryza sativa*	[51]
	Cas12j2	*Pt4CL1*, *PtPDS*, *PtSVP*, *PtMYB182*	1.20%	*Populus tomentosa*	[62]
	Cas12j2	*OsNTTVs*	15–60%	*Oryza sativa*	[62]
	Cas12j2	*SlSGR*, *SlAgo7*, *SlALS*, *SlBlc*	<15%	*Solanum lycopersicum* L.	[62]
	AsCas12f-HKRA	*OsPDS*, *OsYSA*, *OsD14*	27.1%, 24%, 53.1%	*Oryza sativa*	[63]
	SpCas12f	*OsTubulin* (Tub-1, Tub-2)	28.8%, 55.6%	*Oryza sativa*	[64]

## Data Availability

All data are shown in the main manuscript.
